# University of Aberdeen, Scotland

**Published:** 1885-01

**Authors:** Charles W. Purdy


### FOREIGN CORRESPONDENCE.

UNIVERSITY OF ABERDEEN, SCOTLAND.

*My Dear Editor:*—Possibly a few stray notes of observation during my sojourn in the Old World may interest some of your many readers. If, therefore, you do not find your space too much crowded to admit of foreign gossip, I shall be glad during my somewhat extended absence to let you hear from me, as time and opportunity may permit.

Leaving the quaint old city of Quebec, Nov. 15th, clothed in snow and ice, the prospect of a northern voyage across the Atlantic was not an interesting one; yet, somewhat singular for the season, the voyage proved a most charming one, *i. e.*, for the most part clear sky, balmy atmosphere, and remarkably smooth sea. The morning of Nov. 24th found us at the mouth of the Mersey, and as we steamed up its heavy organically-charged waters, I was instinctively reminded of Chicago river. I believe it was Shakspeare who in speaking of the Mersey said, "It is that kind of mercy which is not filtered." Let us hope, however, that the future may reveal a difference in this respect in the two distinguished rivers just named, and that those whose business it shall be in our State to deal with questions of public policy may enact that the Chicago river may at least be drained (if not filtered) "pro bono publico."

Of course every one on landing at Liverpool first runs " up to London," though just why it should be "up" I have not yet satisfied myself. I, therefore, in four and a half hours after landing found myself in the *elevated* metropolis. A preliminary glance over St. Thomas' Hospital in particular and a few other points of interest occupied my stay of two days in London. As I intend to return in January and spend some three months there, I shall reserve what I may have to say of my impressions till more maturely considered.

It was during the week of my visit to London that one of the brightest and most promising lights in medical literature perhaps of the last decade went out so unexpectedly and so early: a deep loss not only to the profession of England but to the world. I refer to the death of Dr. Mahomed, of Guy's Hospital. Only about thirty-five years of age, he had not only made himself widely known through his lucid contributions to current medical literature, but he had also come to be looked upon as one of the almost necessary figures in the noted gatherings of the profession in Europe. His contributions to sphygmographic diagnosis are familiar to most of us. His contributions to the literature of renal diseases (the last, I believe, in Guy's Hospital reports) always so clear, new, and thoroughly original that I have always thought an exceptionally bright professional future was in store for him. When I say that there are few men in Europe with whom I had more pleasurable or profitable anticipations of meeting than Dr. Mahomed on my present visit, it will be more readily understood how sad a disappointment the news of his death came to me.

November 18th finds me in Aberdeen, an old granite city at the foot of the Grampian Hills, almost at the extremity of a peninsula, jutting out into the German sea. At this season of the year scarcely six of the twenty-four hours constituting the day are favored with rays from "old Sol," and these come from a very oblique altitude; for, remember, Aberdeen is situated north of the 57th parallel (almost the same line as the south coast of Norway). The north-east wind from the German sea at this season, as it carries the chilly breath of Norseland with it, I have learned from experience, can pick out an American, as Twain said, "with its eyes shut." Snow, sleet, mists, rains and fogs are the rule, I understand, here till after May, and clear weather the exception. Added to this, the Aberdeen people do not keep their houses warm; in fact, if a fire is used it is made with Scotch coal, and often the outside door is left open a good share of the time. It is not difficult, hence, to understand the most prolific causes which renders the death-rate here so startlingly high in kidney diseases, constituting as it does one forty-ninth of the entire mortality (the highest ratio of any city in the world).

But, if Aberdeen can not boast of her climate in winter, there are yet many things which she may justly regard with pride, and among these not the least is her university, one of the oldest and most honored in the country. A week's experience in this, with the prospect of spending three more here, is my excuse for a general outline of the institution.

The University of Aberdeen derives its origin from two different foundations; one, the University and King's College of Aberdeen, founded in 1494 by William Elphinstone, Bishop of Aberdeen, under the authority of a Papal Bull obtained through the solicitation of King James IV. The other, Marischal College and University of Aberdeen, founded in 1593 by George Keith, Earl Marischal, by a charter ratified by act of Parliament. In 1858 the two institutions were united by act of Parliament, under the style and title of the "University of Aberdeen."

The University is a corporate body, consisting of a chancellor (elected for life)—present one is the Duke of Richmond—rector, principal, professors, registered graduates and alumni and matriculated students. It takes rank among the universities of Scotland, as from date of foundation of the University and King's College, 1494, two years before the discovery of our continent. Aberdeen and Glasgow Universities are the only ones in Scotland entitled to elect a member to serve in Parliament to represent them.

The University buildings, formerly of King's College, are situated in Old Aberdeen, where the classes in Faculties of Arts and Divinity are conducted. The University buildings, formerly of Marischal College, are situated on Broad street, so called (for friends could almost shake hands across it), Aberdeen, where the classes in the Faculties of Law and Medicine are conducted.

The University is sustained by annual payments from crown (grants by King William III out of Bishop's rents) and Parliamentary grants, by rents, interests, teinds, fees, duties; by revenue of trust funds, under charge of the town council of Aberdeen; by matriculation, graduation and other annual fees, and by bursary funds, in all a total annual income of 20,500 pounds sterling. A reserve fund is maintained at present of 2,770 pounds.

The library of the University contains over 90,000 volumes, open to all members and matriculants under reasonable conditions.

The degrees in medicine granted by the University of Aberdeen are: Bachelor of Medicine (four years of medical and surgical study are necessary), Master in Surgery (same time as to study) and Doctor of Medicine. Requirements: Candidates must be an M. B., twenty-four years of age, and serve two years in attendance on a hospital or in military or naval medical service, or in medical or surgical practice *subsequently* to his having received the degree of M. B.

A minute description of the various teaching departments in the medical faculty, much less in the other faculties, would scarcely prove of sufficient interest, perhaps, to excuse its introduction here, even did space and time permit. I shall, therefore, only have a few words to say in reference to the pathological department, the chair of which was furnished by Sir Erasmus Wilson, and is very ably occupied by Professor Hamilton. It is here in the pathological laboratory, reviewing some special point of pathology, that my labors are at present confined almost entirely.

Of course, the facilities for observation and the methods of teaching in Berlin and Vienna are yet in store tor me, and I must not judge too rashly till I have, as I soon expect, an opportunity of testing there. I am aware, also, how popular Vienna is with most of our American physicians as well as students who have visited it.

It is difficult for me, however, to conceive of much greater facilities for pathological study than I find at Aberdeen. Take, for instance, the department which of course interests me most, renal pathology. One is not expected to visit a dead house or to attend a post-mortem. In the pathological laboratory one finds the material in all shapes of preparation, in abundance, at one's finger ends. If he wished to examine the organs as removed from the body, the assistant will bring him almost any member of every known kidney lesion. If he wishes to cut and examine any of these he is at liberty to do so, and, moreover, he will be taught in fifteen minutes how to cut sections ready to mount at the rate of from 100 to 150 in the minute.

If he wishes to study any particular lesion, he will find at his finger ends thousands of cut and ready to mount sections of that lesion in most excellent state of preservation, and all he has to do is to mount and study them. I have yet to name a single lesion of the kidney which I have not found in abundance in the pathological laboratory of Aberdeen, and I have been been on the lookout for many of these myself and among my friends in Chicago for years without success in some cases. Nor is it alone in renal lesions that the material is abundant. Some of my very good friends in Chicago in charge of the nervous system would, I am sure, be surprised at the amount of valuable material in the laboratory at Aberdeen in that line.

In an institution so old, where everything is carefully preserved that can possibly interest or benefit the medical mind, it is but natural that material should become abundant.

The methods of teaching are practical, rapid and wonderfully simple, and what I wish more especially to call attention to is the great consideration and kindness shown to strangers here. The stranger in quest of professional knowledge at Aberdeen will find a special friend in Professor Hamilton. It is often these little courtesies and attentions one meets with from home that broadens one's medical views, and makes us think more of our noble profession; and bearing in mind the characteristic generous-heartedness of our American people, I feel sure that in 1887, when the International Medical College meets in our Capital City, that the profession in America will at least equal their European brethren in one respect, namely, in dispensing a most hearty hospitality.

I had intended to have said something of this city and its surroundings, which are by no means unattractive. The coast scenery is boldly picturesque, with its imposing granite cliffs and boulders; its characteristic Scottish firths, estuaries and fissures, winding within and without the rocky coast line. Nor are the "hills and glens" of which we hear so much in Scottish song less pleasing to the eye, and these are all within easy range, even on foot, from the substantial old city.

But I fear I have trespassed too much on your space and patience, and for the present shall not extend this communication to greater length. Perhaps at Edinburgh or London, my next objective point, I may find something of interest to your readers. CHARLES W. PURDY.

Dec. 6, 1884.

